# Effects of a financial incentive scheme for dementia care on medical and long-term care expenditures: A propensity score–matched analysis using LIFE study data

**DOI:** 10.1371/journal.pone.0282965

**Published:** 2023-03-10

**Authors:** Jun Kawabata, Haruhisa Fukuda

**Affiliations:** 1 Advanced Emergency Medical Service Center, Kurume University Hospital, Kurume, Fukuoka, Japan; 2 Department of Health Care Administration and Management, Graduate School of Medical Sciences, Kyushu University, Fukuoka, Japan; Local Health Authority Caserta: Azienda Sanitaria Locale Caserta, ITALY

## Abstract

**Objective:**

Japan introduced a financial incentive scheme in April 2016 to improve hospital-based dementia care, but its effectiveness remains unclear. This study aimed to investigate the scheme’s impact on medical and long-term care (LTC) expenditures, as well as on changes in care needs levels and daily living independence levels among older persons one year after hospital discharge.

**Methods:**

We linked medical and LTC claims databases, and retrospectively identified patients who received LTC needs certification and daily living independence assessments in Fukuoka, Japan. Case patients (received care under the new scheme) were those admitted from April 2016 to March 2018, and control patients were those admitted from April 2014 to March 2016 (before the scheme was implemented). Through propensity score matching, we identified 260 case patients and 260 control patients, and compared using *t*-tests, and chi-square tests.

**Results:**

The analyses found no significant differences between the case and control groups in medical expenditure (US$26,685 vs US$24,823, *P* = 0.37), LTC expenditure (US$16,870 vs US$14,374, *P* = 0.08), daily living independence level changes (26.5% vs 20.4%, *P* = 0.12), or care needs level changes (36.9% vs 30%, *P* = 0.11).

**Conclusions:**

The financial incentive scheme for dementia care did not demonstrate any beneficial effects on patients’ healthcare expenditures or health conditions. Further studies are needed to examine the scheme’s long-term effects.

## Introduction

The prevalence of dementia is rapidly increasing worldwide [1]. In addition to imposing a considerable financial burden, dementia also places a heavy human cost on patients, caregivers, societies, and countries [2].

Dementia is reportedly common among older persons who are admitted to acute care hospitals [3]. Furthermore, hospitalized patients with dementia are more likely to experience unfavorable outcomes such as functional decline, longer length of stay (LOS), higher readmission rates, and higher mortality rates when compared to those without dementia [4, 5]. Patients with cognitive impairment also have a higher risk of being physically restrained during hospitalization [6], which is associated with poorer outcomes [7–9]. In Japan, population aging had led to an increase in older persons with cognitive impairment or dementia admitted to acute care hospitals [10], and dementia’s societal costs are expected to increase 1.6-fold from 2014 to 2060 [11].

In April 2016, the Japanese government introduced a financial incentive scheme to improve the quality of hospital-based dementia care and patient outcomes by awarding additional benefits to providers for the provision of appropriate care. This scheme was designed to incentivize the provision of multidisciplinary care without the use of physical restraint in acute care hospitals for the management of patients with cognitive impairment. A recent study reported that this scheme was effective in reducing the use of physical restraints [12], but other studies have noted that it has not reduced in-hospital mortality or 30-day readmission rates [13].

Little is known about the impact of this scheme on outcomes such as medical expenditures, long-term care (LTC) expenditures, and patients’ health conditions. This study aimed to investigate the scheme’s possible effects on total medical and LTC expenditures, as well as on changes in care needs levels and daily living independence levels among older persons after being discharged from acute care hospitals. We hypothesized that patients who are treated under this new incentive scheme would have better outcomes than patients not treated under the scheme.

## Materials and methods

### Study design and data source

The Longevity Improvement & Fair Evidence (LIFE) Study is a large-scale multi-regional cohort study that collects and analyzes administrative claims data from National Health Insurance and Later-Stage Elderly Healthcare System enrollees in Japan. Here, we conducted a retrospective observational study using municipal-level LIFE Study data from eight hospitals located in five municipalities within Fukuoka Prefecture. The study data included LTC needs (care needs) certification data, medical claims data, and LTC claims data. Data from April 1, 2014 to March 31, 2019 were obtained. In Japan, all citizens and long-term residents are required to be enrolled in the universal health insurance system, which allows them to receive treatment at any medical institution throughout the country. The medical fees paid to medical institutions by insurers are calculated by adding up the number of service fee points for each medical procedure as determined by the Minister of Health, Labour and Welfare; this payment schedule is revised every two years. In contrast, LTC fees are paid to service providers for the provision of nursing care services to users with certified care needs, and this payment schedule is revised every three years. Our medical claims data and LTC claims data included the reimbursement claims from providers to the municipal governments, which fulfill the role of insurer for National Health Insurance and Later-Stage Elderly Healthcare System enrollees.

Our analysis was designed to investigate the effects of the financial incentive scheme for dementia care that was introduced on April 1, 2016. This scheme provides financial incentives for hospitals to provide two types of dementia care: Type 1 care involves higher financial incentives, and requires the presence of a dementia-specific multidisciplinary team comprising physicians trained in dementia management, advanced practice nurses trained in dementia or geriatric care, and psychiatric social workers. Type 2 care involves lower financial incentives, but only requires two or more nurses trained in the assessment and care of patients with dementia. Under this scheme, the government pays hospitals an additional US$14.5/day and US$2.9/day (US$1 = \103.412 in January 2020) for each eligible patient given Type 1 care and Type 2 care, respectively; this incentive is reduced to US$2.9/day and US$0.97/day, respectively, after 15 days from the start of care by a dementia care specialist team. However, the incentive is reduced to 60% if physical restraints are used. Based on a preliminary analysis of the LIFE Study data, we chose the eight target hospitals due to their high numbers of patients who had received care under this scheme.

### Study patients

The study population was divided into a control group and a case group. The control group consisted of patients who had been admitted between April 1, 2014 and March 31, 2016 (i.e., before the incentive scheme was implemented). The case group consisted of patients who had been admitted between April 1, 2016 and March 31, 2018 (i.e., after the incentive scheme was implemented), and had records of being treated under this incentive scheme. To eliminate hospital-specific bias, each control patient was selected from the same hospital as their matching case patient.

We first selected patients who had received assessments for daily living independence level of older persons with dementia [14] and care needs certification. Because, these assessments have been recorded daily living independence level of older persons with dementia. The incentive scheme is individually applied to eligible patients with some degree of functional disability of daily living independence level. We focused on patients who could be tracked for one year starting from the month after hospital discharge. Patients were excluded if they had died during hospitalization, had an LOS of only one day, or had died within one year after discharge.

Each patient’s degree of dementia severity was assessed using his/her daily living independence level of older persons with dementia. These levels were categorized into the following grades based on functional capacity in activities of daily living (ADL): independent (no dementia symptoms and functionally independent), Grade I (dementia symptoms with no ADL limitations), Grade IIa (dementia symptoms, able to live independently with some assistance when outdoors, and can manage one’s own medication), Grade IIb (dementia symptoms, able to live independently with some assistance at home, but cannot manage one’s own medication), Grade IIIa (dementia symptoms; moderate ADL limitations due to symptoms, behavior, and/or communication difficulties; and requires daytime assistance), Grade IIIb (dementia symptoms, moderate ADL limitations, and requires nighttime assistance), Grade IV (dementia symptoms, severe ADL limitations, and requires full-time care), or Grade M (dementia symptoms, and requires full-time care for severe psychiatric symptoms and/or severe physical disorders including delirium). The incentive scheme is individually applied to eligible patients with some degree of functional disability in ADL (Grades IIIa, IIIb, IV, or M) without severely impaired consciousness. Eligibility was evaluated based on the identification of specific dementia symptoms (irrespective of whether a clinical diagnosis of dementia was given) and the need for treatment in a specialized facility due to severe mental disorders or physical illness.

Under Japan’s LTC insurance system, certified care needs are categorized into seven levels (support levels 1–2 and care needs levels 1–5), where higher levels indicate more severe disabilities with greater requirements for LTC.

### Outcome measures

The primary outcome measures were the total medical expenditure and total LTC expenditure for one year after discharge. The secondary outcome measures were the deterioration of daily living independence levels and care needs levels one year after discharge. The deterioration of levels in the secondary outcome measures was analyzed as increases of one level or more using dummy binary variables (increase of ≥1 level vs. no increase). The outcome measures were compared between the case and control groups. For patients who did not receive an update or reapplication for the level of care needs within 12 months of discharge, we assumed that their care needs levels had remained unchanged during that period. Because care needs certification re-assessments are only conducted every two years, not all patients would have undergone these evaluations during the first year after discharge.

### Covariates

The covariates included sex, age, Charlson Comorbidity Index (CCI) score, LOS, hospitalization expenditures, daily living independence level on admission, and care needs level on admission. CCI scores were included because higher scores are associated with higher annual medical and LTC expenditures among older patients [15].

LOS and hospitalization expenditures were included as potential indicators of more severe illness.

### Statistical analysis

First, we linked the medical claims data and LTC claims data for individual patients based on LTC certification evaluations from each municipality using patient identification numbers assigned for research purposes. Next, each case patient was matched with a control patient from the same hospital using propensity scores (PS). Finally, we combined the datasets of the PS-matched models from the eight hospitals in five municipalities.

The following covariates were entered into logistic regression models to calculate the PS for receiving care under the incentive scheme: sex (male/female), age (continuous variable), CCI score (continuous variable), LOS (continuous variable), hospitalization expenditures (continuous variable), daily living independence level on admission (categorical variable), and care needs level on admission (categorical variable). For the PS model, care needs level on admission was categorized into two groups (i.e., a dummy binary variable): a lower level (from support level 1 to care needs level 2) and a higher level (care needs levels 3 to 5). One-to-one nearest-neighbor matching without replacement was conducted using a caliper width of 0.2 standard deviations of the logit of the estimated PS. The discriminatory power (predictive accuracy) of each logistic regression model was evaluated with the c-statistic. Using these methods, we combined the datasets before and after PS matching in the five municipalities.

We used the *t*-test and Mann–Whitney *U* test to compare the averages of the continuous variables (i.e., total medical and LTC expenditures) and the chi-square test to compare the proportions of the categorical variables (i.e., deterioration of daily living independence levels and care needs levels) between the case and control groups. The associations between the implementation of the incentive scheme and the study outcomes were first examined with these univariable analyses. Thereafter, the continuous variables were analyzed using multivariable linear regression models, and the categorical variables were analyzed using multivariable logistic regression models. For the logistic regression models, the dependent variables (i.e., deterioration of daily living independence levels and care needs levels) were analyzed as increases of ≥1 level (represented by a value of 1) vs. no increase (represented by a value of 0). The coefficients, odds ratios (ORs), and 95% confidence intervals (CIs) were calculated.

All analyses were conducted using R version 3.0.4, and the threshold for significance was *P* < 0.05 (two-tailed).

This study was approved by the Institutional Review Board of Kyushu University (Approval No. 2019–045). The requirement for informed consent was waived by the review board due to the retrospective nature of the study, and also because all records were de-identified and fully anonymized before we accessed them for analysis.

## Results

### Study patients

**Fig 1:**
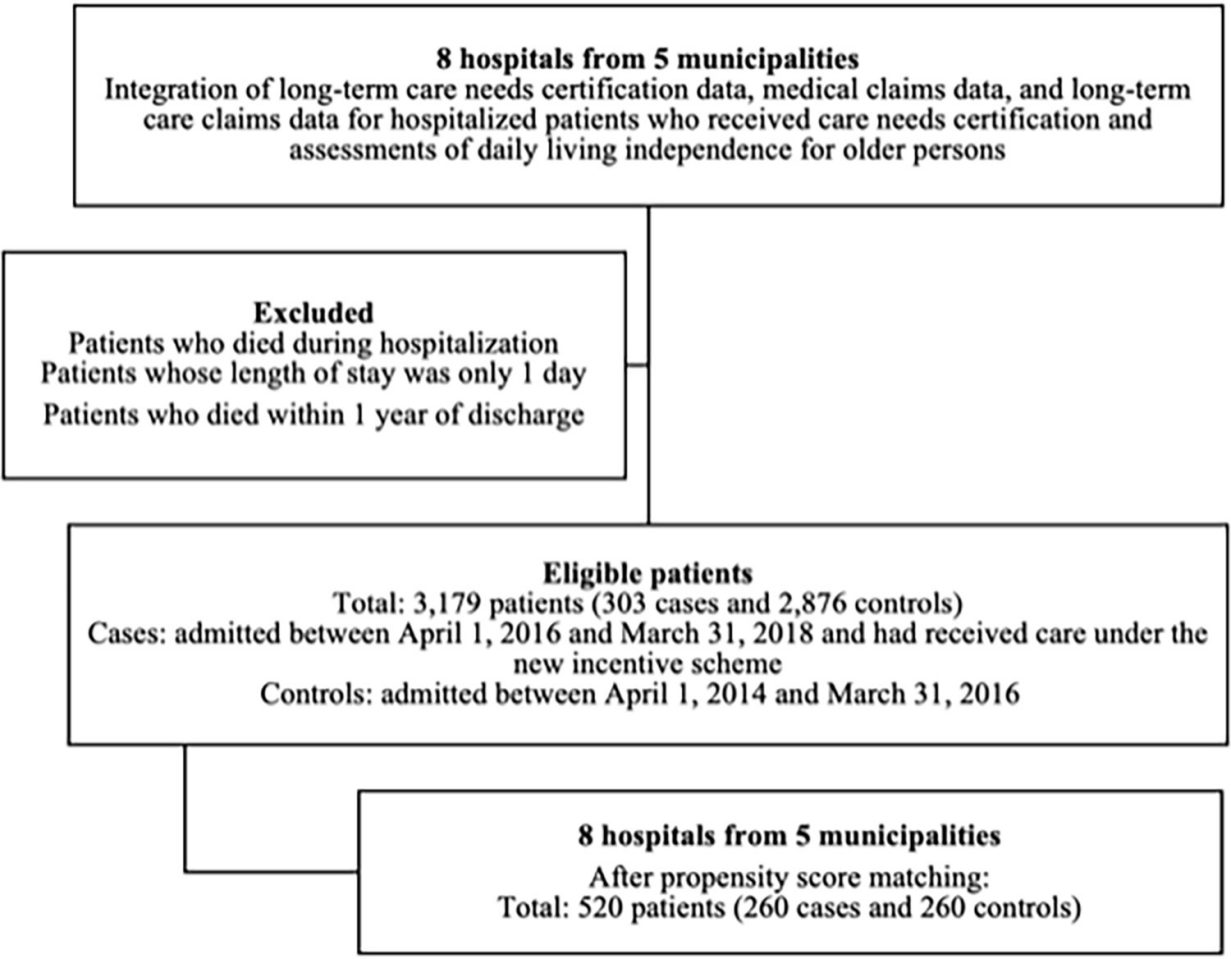
Patient selection process. Propensity score matching was conducted based on sex, age, Charlson Comorbidity Index score, length of stay, hospitalization expenditures, daily living independence level, and care needs level.

The patient selection process is presented in Fig 1.

Table 1 summarizes the baseline characteristics of the case and control patients before and after PS matching. A total of 3,179 eligible patients (303 case patients and 2,876 control patients) were initially identified. In this study, patients were only provided with Type 2 care under the incentive scheme. After PS matching, we obtained a total of 520 patients (260 case patients and 260 control patients) from eight hospitals across five municipalities. We excluded 43 cases as they could not be matched with any controls under the specified matching criteria. The mean c-statistic of the regression models for calculating PS was 0.82 (95% CI: 0.74–0.89).

**Table 1:** Patient characteristics before and after PS matching.

	Before PS Matching (n = 3,179)			After PS Matching (n = 520)		
Characteristics	Case Group	Control Group	SMD	Case Group	Control Group	SMD
	n = 303	n = 2,876		n = 260	n = 260	
**Female, n (%)**	199 (65.7)	1,781 (61.9)	0.71	165 (63.5)	163 (62.7)	0.02
**Age (years), mean (SD)**	86.6 (6.6)	82.9 (7.9)	0.51	86.1 (6.6)	85.8 (7.5)	0.04
**CCI score, mean (SD)**	3.5 (2.4)	3.2 (2.4)	0.13	3.5 (2.4)	3.4 (2.5)	0.02
**Length of stay (days), mean (SD)**	27.2 (23.3)	24.6 (41)	0.08	26.8 (22.9)	28 (30)	0.05
**Length of stay (days), median [IQR]**	21 [13, 34]	16 [8, 30]	0.08	20 [12, 33]	18 [9, 36]	0.05
**Hospitalization expenditures (USD), mean (SD)**	1,208 (1,131)	1,156 (2,383)	0.03	1,184 (1,058)	1,244 (1,275)	0.05
**Hospitalization expenditures (USD), median [IQR]**	868 [545, 1,532]	686 [352, 1,432]	0.03	832 [543, 1,537]	783 [438, 1,588]	0.05
**Daily living independence level on admission, n (%)**			0.56			0.12
**Independent**	16 (5.3)	515 (17.9)		16 (6.2)	18 (6.9)	
**Grade I**	38 (12.5)	559 (19.4)		34 (13.1)	34 (13.1)	
**Grade IIa**	32 (10.6)	380 (13.2)		31 (11.9)	26 (10.0)	
**Grade IIb**	59 (19.5)	505 (17.6)		52 (20.0)	57 (21.9)	
**Grade IIIa**	75 (24.8)	426 (14.8)		59 (22.7)	55 (21.2)	
**Grade IIIb**	31 (10.2)	170 (5.9)		24 (9.2)	20 (7.7)	
**Grade IV**	48 (15.8)	263 (9.1)		40 (15.4)	44 (16.9)	
**Grade M**	4 (1.3)	58 (2.0)		4 (1.5)	6 (2.3)	
**Care needs level on admission**	150 (49.5)	1140 (39.6)	0.2	124 (47.7)	125 (48.1)	0.01
**(Care needs levels 3–5), n (%)**						

PS, propensity score; SMD, standardized mean difference; SD, standard deviation; CCI, Charlson comorbidity index; IQR, interquartile range.

### Comparison of outcomes

Table 2 presents the results of the univariable comparisons of outcomes between the case and control groups. There were no significant differences in total medical expenditure between the case and control groups according to the *t*-test (US$26,685 vs US$24,823, *P* = 0.37) and Mann–Whitney *U* test (US$21,438 vs US$17,245, *P* = 0.11). Although not significant, patients in the case group tended to have higher total LTC expenditure than those in the control group (*t*-test: US$16,870 vs US$14,374, *P* = 0.08; Mann–Whitney *U* test: US$13,308 vs US$8,539, *P* = 0.07). The chi-square test showed no significant difference between the case and control groups in the deterioration of daily living independence levels (increase of ≥1 levels: 26.5% vs 20.4%, *P* = 0.12) or care needs levels (increase of ≥1 levels: 36.9% vs 30%, *P* = 0.11).

**Table 2:** Univariable analyses of outcomes between the PS-matched case and control patients.

	Case Group	Control Group		
	n = 260	n = 260	*P* value	Statistical test
**Total medical expenditure (USD), mean (SD)**	26,685 (22,587)	24,823 (24,313)	0.37	*t*-test
**Total LTC expenditure (USD), mean (SD)**	16,870 (16,566)	14,374 (16,090)	0.08	
**Total medical expenditure (USD), median [IQR]**	21,438 [8,402, 37,026]	17,245 [6,328, 34,622]	0.11	Mann–Whitney *U* test
**Total LTC expenditure (USD), median [IQR]**	13,308 [1,357, 27,506]	8,539 [0, 26,258]	0.07	
**Daily living independence level deterioration (increase of ≥1 level), n (%)**	69 (26.5)	53 (20.4)	0.12	Chi-square test
**Care needs level deterioration (increase of ≥1 level), n (%)**	96 (36.9)	78 (30)	0.11	

PS, propensity score; IQR, interquartile range; LTC, long-term care; SD, standard deviation.

Table 3 shows the results of the multivariable linear regression analyses with total medical expenditure and total LTC expenditure for one year after discharge as the dependent variables. The incentive scheme was neither associated with total medical expenditure (Coef. US$2,473, 95% CI: -1,135–6,081, *P* = 0.18) nor with total LTC expenditure (Coef. US$2,169, 95% CI: -520–4,858, *P* = 0.11). However, total medical expenditure was significantly associated with LOS (Coef. US$170, 95% CI: 65–275, *P* = 0.002) and hospitalization expenditures (Coef. US$0.05, 95% CI: 0.02–0.07, *P* < 0.001).

**Table 3:** Multivariable linear regression analyses of total medical and LTC expenditures for one year after discharge.

Variables	Total Medical Expenditure (USD)			Total LTC Expenditure (USD)		
	Estimate	95% CI	*P* value	Estimate	95% CI	*P* value
**Use of incentive scheme for dementia care (ref: non-use)**	2,473	-1,135, 6,081	0.18	2,169	-520, 4,858	0.11
**Male (ref: female)**	1,027	-2,931, 4,985	0.61	-4,133	-7,083, -1,184	0.006
**Age**	-394	-663, -126	0.004	470	270, 670	<0.001
**Charlson Comorbidity Index score**	63	-688, 815	0.87	-336	-896, 224	0.24
**Length of stay**	170	65, 275	0.002	13	-65, 91	0.74
**Hospitalization expenditures**	0.05	0.02, 0.07	<0.001	-0.002	-0.019, 0.015	0.83
**Daily living independence level on admission (ref: independent)**						
**Grade I**	-6,846	-15,481, 1,790	0.12	4,703	-1,733, 11,139	0.15
**Grade IIa**	-12,790	-21,735, -3,844	0.005	8,212	-1,545, 14,879	0.016
**Grade IIb**	-9,991	-18,097, -1,884	0.016	7,814	1,773, 13,855	0.012
**Grade IIIa**	-10,933	-19,097, -2,769	0.009	9,729	-3,645, 15,813	0.002
**Grade IIIb**	-8,588	-18,226, 1,051	0.081	12,378	5,195, 19,562	<0.001
**Grade IV**	-15,143	-23,976, -6,309	<0.001	7,874	1,291, 14,457	0.019
**Grade M**	-15,509	-30,332, -686	0.041	6,740	-4,307, 17,787	0.23
**Care needs level on admission**	-1,719	-5,867, 2,429	0.42	2,121	-971, 5,212	0.18
**(Care needs levels 3–5; ref: support level 1 to care needs level 2)**						

LTC, long-term care; CI, confidence interval.

Table 4 shows the results of the multivariable logistic regression analyses with the deterioration of care needs levels and daily living independence levels at one year after discharge as the dependent variables. The incentive scheme was neither associated with daily living independence levels (OR: 1.51, 95% CI: 0.96–2.38, *P* = 0.07) nor with care needs levels (OR: 1.44, 95% CI: 0.97–2.16, *P* = 0.07).

**Table 4:** Multivariable logistic regression analyses of deterioration in care needs levels and daily living independence levels at one year after discharge.

Variables	Daily Living Independence Level Deterioration^a^			Care Needs Level Deterioration^a^		
	OR	95% CI	*P* value	OR	95% CI	*P* value
**Use of incentive scheme for dementia care (ref: non-use)**	1.51	0.96, 2.38	0.07	1.44	0.97, 2.16	0.07
**Male (ref: female)**	0.83	0.50, 1.36	0.46	1.09	0.70, 1.68	0.71
**Age**	1.02	0.99, 1.06	0.26	1.05	1.02, 1.08	0.003
**Charlson Comorbidity Index score**	1.05	0.96, 1.15	0.27	1.03	0.95, 1.11	0.51
**Length of stay**	0.99	0.98, 1.01	0.21	1	0.99, 1.01	0.76
**Hospitalization expenditures**	1	1.00, 1.00	0.11	1	1.00, 1.00	0.062
**Daily living independence level on admission (ref: independent)**						
**Grade I**	0.51	0.22, 1.19	0.12	1.21	0.50, 2.96	0.68
**Grade IIa**	0.57	0.23, 1.36	0.21	1.07	0.43, 2.72	0.88
**Grade IIb**	0.32	0.14, 0.71	0.006	1.29	0.56, 3.05	0.55
**Grade IIIa**	0.12	0.05, 0.30	<0.001	0.92	0.39, 2.21	0.85
**Grade IIIb**	0.21	0.07, 0.60	0.004	1.18	0.41,3.35	0.76
**Grade IV**	0	0	0.98	0.76	0.27, 2.10	0.6
**Grade M**	NA	NA	0.99	0.48	0.06, 2.54	0.42
**Care needs level on admission**	0.64	0.38, 1.05	0.08	0.19	0.12, 0.31	<0.001
**(Care needs levels 3–5; ref: support level 1 to care needs level 2)**						

OR, odds ratio; CI, confidence interval.

^a^ Deterioration was analyzed as increases of ≥1 level (represented by a value of 1) vs. no increase (represented by a value of 0).

## Discussion

This study investigated the potential beneficial effects of a financial incentive scheme aimed at improving dementia care in Japanese hospitals. Our results indicated that the scheme was not significantly associated with patients’ healthcare expenditures or health conditions one year after hospital discharge. However, LOS and hospitalization expenditures were found to be positively associated with total medical expenditure in the year after discharge, suggesting that advanced medical care and disease severity are important factors when considering the post-discharge financial burden of patients.

Our findings that this incentive scheme was not associated with improved outcomes are consistent with those of previous studies [12, 13]. However, one study had included a sensitivity analysis that was restricted to patients who were treated by a dementia specialist team, and the incentive scheme was found to be associated with reduced in‐hospital mortality under those conditions [13]. In addition, a national cross-sectional study found that the incentive was able to reduce the use of physical restraints for patients with dementia [11].

In our PS-matched analysis, we found no significant differences between the case and control groups in healthcare expenditures, daily living independence levels, and care needs levels one year after discharge. Studies have reported that appropriate dementia care can reduce the risk of adverse patient outcomes and the cost of acute dementia care [16, 17]. Further research is therefore needed to determine if comprehensive dementia care can help to improve outcomes in patients with dementia. However, we should also consider the possibility that the number of matched cases was too small and that the study was under-powered. This may have contributed to the lack of significant differences in these outcomes.

The main findings of our study are as follows. First, the incentive scheme was not associated with healthcare expenditures or health conditions. The scheme was only introduced in 2016, and dementia care education in acute care hospitals may still be underdeveloped. Although various approaches to dementia care education have been proposed [18], there is currently no standardized best practice. A one-sample descriptive correlation study reported that nurses working in acute care settings had moderate competence in providing dementia care, with inadequate knowledge of its special requirements [19]. Hence, hospitals may still have problems in providing effective and appropriate dementia care. In addition, our study patients only included those who were treated with Type 2 care. These results suggest that the incentive scheme in its current form is not working effectively. Even though the financial incentive is substantially reduced if physicians and nurses decide to use physical restraints, this condition did not appear sufficient to affect the decision-making process. Further adjustments may be needed to improve the incentive scheme and reduce the use of physical restraints.

Second, the case group tended to have higher expenditures and higher levels of deterioration than the control group. Although our results were contrary to the hypothesis, this observation may have been because the overall healthcare expenditures before death can vary with age and care [20]. Therefore, the date of death should be considered an important factor in the follow-up of patients. Furthermore, our study did not account for variations in disease severity among the patients. It is possible that the case group included a larger proportion of severely ill patients, which manifested as a higher need for medical and LTC resources after discharge.

Third, LOS and hospitalization expenditures were positively associated with total medical expenditure during the first year after hospital discharge. Older persons with cognitive impairment may experience further cognitive decline after being discharged [21], which may have influenced the observed increases in post-discharge medical expenditure. Furthermore, we postulate that severely ill patients may also have had an impact on post-discharge medical expenditure as these patients would have more prolonged LOS and higher hospitalization expenditure than those with less severe disease.

Fourth, the total medical and LTC expenditures were not associated with the CCI score. These results contrasted with those of a previous report that showed an association between multimorbidity and healthcare expenditures in older persons [22]. The disparity in findings may be because we did not adjust for the severity of each comorbidity in our subjects.

Several limitations should be considered when interpreting our results. First, we could not adjust for stroke, dementia, or other diseases, which would likely affect patients’ healthcare expenditures and health conditions. Nevertheless, we were able to adjust for the patients’ CCI scores and daily living independence levels as part of their baseline characteristics. Second, our follow-up period was only one year. As dementia is a chronic disease, it would be more advantageous to evaluate the scheme’s impact over a longer duration, such as in a previous study that used a 4- to 5-year follow-up period [23]. Third, we were unable to adjust for each patient’s household income and family structure. A previous retrospective cohort study evaluated the use of medical and LTC services by older persons with dementia according to their income levels [24]. Older persons from lower-income households may be more reluctant to use home rehabilitation services [25], and those who live alone may be more likely to choose hospital or nursing home admission due to the lack of family caregivers. The influence of household income and other factors should therefore be considered when estimating medical and LTC expenditures. Fourth, the study was conducted in five municipalities in Fukuoka Prefecture, and the results may have limited external validity and generalizability. It is therefore important to conduct similar analyses in other regions. Moreover, data from the eight hospitals were combined after PS matching for each hospital. Although the inclusion of hospital fixed effects in the regression analyses could have helped to account for institutional variations in procedures, this was precluded by the small sample size of patients in the case group. Fifth, the incentive seems to focus on providing quality care during the admission of the patients. The outcomes one year after hospital discharge used in this study would be affected by various factors such as types and the quality of home care services after discharge, rather than the in-admission care. Post-discharge patient events also need to be considered.

Patients with cognitive impairment and dementia require high-quality care and management without unnecessary physical restraint. It has been reported that older patients with dementia tend to experience declining physical and cognitive functions when hospitalized [21]. Since physical restraint is associated with delirium [9], minimizing its use could reduce the incidence of this cognitive disorder. Studies should be conducted to examine the incentive scheme’s effect on this outcome.

## Conclusion

The financial incentive scheme for dementia care was not associated with reductions in total medical and LTC expenditures, nor was it associated with one-year changes in care needs and daily living independence levels. Our findings may contribute to the further evaluation and improvement of this incentive scheme, thereby increasing the quality of dementia care in Japan’s acute care hospitals. It is important to improve the multiple medical professions engagement and education involved in care. It is necessary to evaluate and improve the contents of the incentive scheme for dementia care while assessing middle to long-term patient outcomes.
